# Research Progress on the Pathogenesis and Diagnostic Biomarkers of Azoospermia

**DOI:** 10.3390/biom16060877

**Published:** 2026-06-15

**Authors:** Jiazhen Zou, Huihui Gao, Qingdan Gu, Peng Zhang, Heran Cao

**Affiliations:** 1 Department of Laboratory Medicine, Southern University of Science and Technology Yantian Hospital, Shenzhen 518000, China; 2022050403@nwafu.edu.cn (J.Z.); 2025390069@gzhmu.edu.cn (Q.G.); 2 Department of Obstetrics and Gynecology, The Central Hospital of Wuhan, Tongji Medical College, Huazhong University of Science and Technology, Wuhan 430014, China; berylgao@nwafu.edu.cn; 3 Institute of Reproductive Health, Tongji Medical College, Huazhong University of Science and Technology, Wuhan 430030, China; 4 NHC Key Laboratory of Male Reproduction and Genetics, Guangdong Provincial Reproductive Science Institute, Guangzhou 510600, China

**Keywords:** azoospermia, non-obstructive azoospermia, obstructive azoospermia, biomarkers, small RNAs, proteomics, diagnosis, pathogenesis

## Abstract

Azoospermia represents the most severe manifestation of male infertility and is classified into obstructive azoospermia (OA) and non-obstructive azoospermia (NOA). NOA patients experience a lack of sperm due to testicular dysfunction, posing significant challenges in clinical diagnosis and treatment. Recent advancements in molecular biology and high-throughput technologies have led to the discovery and validation of numerous biomarkers, including proteins, non-coding RNAs, genetic polymorphisms, and imaging indicators, which have greatly enhanced the understanding of the pathophysiological mechanisms of azoospermia and facilitated non-invasive diagnostic approaches. This review aims to systematically summarize the pathogenesis of azoospermia and critically evaluate the latest advancements in diagnostic and prognostic biomarkers, including small RNAs, proteomic profiles, genetic markers, and imaging features. The overarching goal is to synthesize this knowledge toward the development of integrated, biomarker-guided strategies for precise diagnosis, prognosis prediction, and improved clinical management of azoospermia, particularly NOA.

## 1. Introduction

Azoospermia, defined as the complete absence of sperm in the ejaculate, is a significant cause of male infertility, affecting approximately 1% of the male population and accounting for 10–15% of all infertility cases [1]. It is categorized into two main types: obstructive azoospermia (OA), where sperm production occurs but is obstructed from being released [2], and non-obstructive azoospermia (NOA), where there is a failure in sperm production due to testicular dysfunction [3]. The distinction between these two types is crucial as it influences the management and treatment options available for affected individuals [4]. Understanding the underlying mechanisms and exploring potential diagnostic biomarkers for azoospermia is essential for improving reproductive outcomes for men facing this condition. The epidemiological significance of azoospermia in male infertility is profound, with studies indicating that a considerable proportion of men diagnosed with infertility present with this condition [5]. Recent findings suggest that genetic factors contribute significantly to the pathogenesis of azoospermia, with many cases remaining idiopathic even after extensive evaluation [3]. This highlights the urgent need for improved diagnostic tools that can accurately classify azoospermia and guide treatment decisions. Traditional diagnostic methods, such as testicular biopsy, while informative, are invasive and carry risks that may deter patients from undergoing necessary evaluations. Therefore, there is a growing emphasis on the development of non-invasive biomarkers that could facilitate the diagnosis of azoospermia types, thus enhancing patient management [6].

The limitations of conventional diagnostic approaches underscore the necessity for innovative solutions in the field of male infertility. Current methods, including hormonal assessments and semen analysis, often fall short in providing a comprehensive understanding of the underlying causes of azoospermia [7]. Invasive procedures such as testicular biopsy, while valuable, can lead to complications and do not always yield conclusive results. As such, the exploration of biomarkers, particularly those derived from seminal plasma or circulating extracellular vesicles, presents a promising avenue for enhancing diagnostic accuracy [8]. Studies have identified various proteins and microRNAs that may serve as potential biomarkers for differentiating between OA and NOA, allowing for more tailored treatment strategies [9,10,11].

This narrative review employed a systematic literature search strategy to ensure comprehensiveness. We searched the PubMed and Web of Science databases, restricting the publication date range from 2015 to 2025. The keyword combinations included: “azoospermia”, “non-obstructive azoospermia (NOA)”, “obstructive azoospermia (OA)”, “biomarkers”, “pathogenesis”, combined with “diagnosis”, “small RNAs”, “microRNA”, “tsRNA”, “piRNA”, “proteomics”, “lncRNA”, “gene polymorphisms”, “imaging”, and “machine learning”, among others. The inclusion criteria were: (1) original research, preclinical and clinical studies exploring the pathogenesis, diagnostic biomarkers, or treatment prognosis of azoospermia, especially NOA; (2) review and research articles involving the application and validation of small RNAs, proteins, gene expression, imaging, or computational models in azoospermia; (3) peer-reviewed English publications. Exclusion criteria comprised non-English publications, conference abstracts, editorials, commentaries, and studies not directly relevant to the core topic. Through screening titles, abstracts, and full texts, 93 studies were finally included and integrated to systematically outline the latest advances in the pathogenesis and diagnostic biomarkers of azoospermia, particularly non-obstructive azoospermia.

In light of these challenges, this review has three aims: (1) to summarize the current understanding of the pathogenesis underlying azoospermia, focusing on NOA; (2) to critically evaluate the latest advancements in diverse diagnostic and prognostic biomarkers, encompassing molecular (small RNAs, proteins, genes), imaging, and computational predictors; and (3) to discuss how the integration of this multi-dimensional knowledge can inform the development of non-invasive diagnostic pathways and personalized management strategies. By synthesizing recent research, we aim to provide a comprehensive framework that links molecular mechanisms to clinical applications.

## 2. Pathophysiological Mechanisms of Azoospermia

### 2.1. Causes and Mechanisms of Obstructive Azoospermia

**Figure 1:**
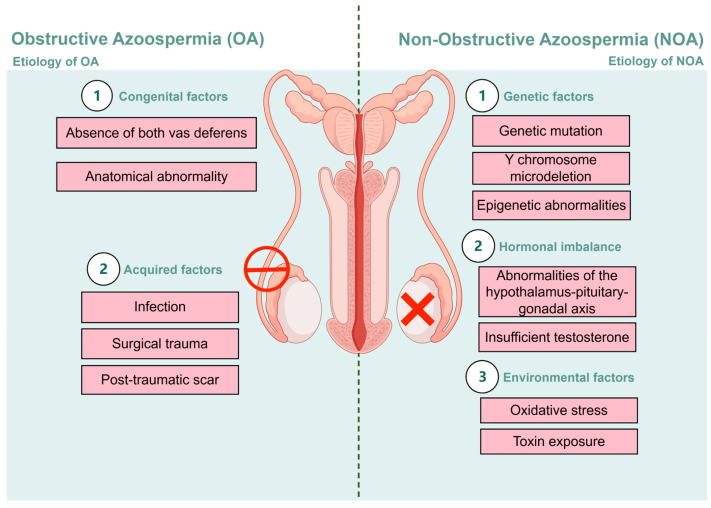
Etiological Classification of Azoospermia (created with Figdraw, www.figdraw.com accessed on 15 August 2025). This figure provides a schematic representation of the two main etiological categories of azoospermia: Obstructive Azoospermia (OA) and Non-Obstructive Azoospermia (NOA). The left panel illustrates OA, characterized by normal spermatogenesis but a physical obstruction preventing sperm transport. Etiologies are subdivided into congenital factors and acquired factors The right panel illustrates NOA, characterized by primary testicular dysfunction impairing sperm production. Etiologies are categorized into genetic factors, hormonal imbalances, and environmental factors. OA, Obstructive Azoospermia; NOA, Non-Obstructive Azoospermia.

Obstructive azoospermia (OA) is characterized by the presence of sperm production in the testes, yet the sperm fails to reach the ejaculate due to blockages in the male reproductive tract. The types of obstruction can vary widely and may include congenital anomalies, such as bilateral absence of the vas deferens, or acquired conditions resulting from infections, trauma, or surgical interventions [12]. For example, conditions like epididymitis or vasectomy can lead to scarring and obstruction [13]. The underlying pathophysiological mechanisms often involve inflammation, fibrosis, and anatomical malformations that impede the normal transport of sperm from the testes through the epididymis and into the ejaculate [14]. In cases of congenital absence, such as in the case of congenital bilateral absence of the vas deferens (CBAVD), the obstruction is due to developmental issues that prevent the formation of the vas deferens entirely, leading to a complete absence of sperm in the ejaculate despite normal spermatogenesis [12,15] (Figure 1).

The pathological characteristics of obstructive azoospermia also reveal that while sperm production may be normal, the transport mechanism is disrupted. This can be observed through imaging studies, such as ultrasound or magnetic resonance imaging (MRI), which can identify structural abnormalities in the reproductive tract [16,17]. For instance, in cases of epididymal obstruction, the epididymis may appear enlarged or show signs of inflammation, which can be indicative of an obstructive process [18]. Furthermore, the histological examination of testicular biopsies in OA patients often shows normal or near-normal spermatogenesis, distinguishing it from non-obstructive azoospermia (NOA), where spermatogenic failure is typically evident.

The differentiation between obstructive and non-obstructive azoospermia is crucial, as it influences the management and treatment options available to affected individuals. Surgical interventions, such as vasoepididymostomy [19] or vasovasostomy [2], can restore fertility in cases of obstructive azoospermia, provided that the obstruction is identified and addressed appropriately. The identification of specific biomarkers in seminal plasma, such as proteins and microRNAs, is also being explored as a non-invasive method to distinguish between obstructive and non-obstructive causes of azoospermia, which could enhance diagnostic accuracy and guide treatment strategies [20,21]. Overall, understanding the causes and mechanisms of obstructive azoospermia is essential for developing targeted therapies and improving reproductive outcomes for affected men.

### 2.2. The Etiology and Mechanisms of Non-Obstructive Azoospermia

NOA is a complex male infertility condition characterized by the complete absence of sperm in the ejaculate due to intrinsic testicular dysfunction. The etiology of NOA is multifaceted, with testicular dysfunction being a leading cause. One primary mechanism involves defects in spermatogenesis, which can arise from various factors, including genetic abnormalities, hormonal imbalances, and environmental influences. Genetic factors such as mutations in specific genes related to spermatogenesis, such as those affecting the synaptonemal complex, have been implicated in cases of meiotic arrest and Sertoli cell-only syndrome, both of which are common histological findings in NOA patients [22]. Furthermore, hormonal dysregulation, including abnormalities in the hypothalamic-pituitary-gonadal axis, can lead to insufficient testosterone production, further impairing spermatogenesis [3]. The interplay between these genetic and hormonal factors creates a challenging landscape for understanding and diagnosing NOA, necessitating a comprehensive approach to patient evaluation and management.

Factors such as oxidative stress, inflammation, and disrupted microenvironments within the testes can exacerbate this apoptotic process, creating a vicious cycle that further impairs spermatogenesis [23,24]. Additionally, the testicular microenvironment, including the composition of testicular interstitial fluid (TIF), has been shown to play a crucial role in supporting germ cell survival and maturation. Alterations in the protein composition of TIF have been associated with defective spermatogenesis, highlighting the importance of local factors in the etiology of NOA [25].

Immune cell infiltration and autoimmune responses also contribute to the etiology of NOA. In some patients, the presence of immune cells within the testes can lead to an inflammatory response that disrupts normal spermatogenesis. This immune-mediated damage can result in the production of autoantibodies against sperm antigens, further exacerbating the infertility condition [26]. The relationship between inflammation and spermatogenesis is complex, as inflammatory cytokines can induce apoptosis in germ cells while also affecting the function of Sertoli cells, which are vital for supporting spermatogenesis [27]. In conclusion, the etiology of non-obstructive azoospermia is multifactorial, involving a combination of genetic, hormonal, and environmental factors that lead to impaired spermatogenesis (Figure 1). The mechanisms underlying NOA include testicular dysfunction, increased germ cell apoptosis, alterations in the testicular microenvironment, and immune-mediated damage.

### 2.3. The Role of Genetic Factors in Azoospermia

The genetic underpinnings of azoospermia, particularly NOA, have garnered significant attention in recent research, highlighting the intricate relationship between genetic factors and male infertility. One of the most critical genetic contributors to azoospermia is the presence of Y chromosome microdeletions, particularly in the azoospermia factor (AZF) regions, such as AZFa, AZFb, and AZFc. These microdeletions can lead to severe spermatogenic impairment, resulting in the complete absence of sperm in the ejaculate. Studies have shown that men with AZF microdeletions have a markedly reduced chance of sperm retrieval during assisted reproductive techniques, underscoring the importance of genetic screening in this population [28,29]. Additionally, specific polymorphisms in key genes related to spermatogenesis, such as SYCP3, TNP2, and DDX3Y, have been implicated in the pathogenesis of azoospermia. Variations in these genes can disrupt normal spermatogenic processes, leading to impaired sperm production. For instance, mutations in the SYCP3 gene, which plays a vital role in the formation of the synaptonemal complex during meiosis, have been associated with meiotic arrest and subsequent azoospermia [30]. Furthermore, the expression of these genes can be influenced by epigenetic modifications, which add another layer of complexity to the genetic landscape of azoospermia. Abnormal gene expression patterns, often resulting from environmental factors or lifestyle choices, can further exacerbate spermatogenic dysfunction [31,32].

In addition to Y chromosome microdeletions and gene polymorphisms, the role of gene expression abnormalities and epigenetic regulation is becoming increasingly recognized in the context of azoospermia. Epigenetic modifications, such as DNA methylation and histone modifications, can significantly impact gene expression without altering the underlying DNA sequence. These modifications can be influenced by various factors, including environmental exposures, nutritional status, and lifestyle choices, potentially leading to altered spermatogenesis and infertility [32]. Recent studies have identified specific epigenetic markers associated with impaired spermatogenesis, suggesting that epigenetic regulation may serve as a potential therapeutic target in the management of azoospermia. For instance, the identification of aberrant DNA methylation patterns in genes critical for spermatogenesis could pave the way for novel diagnostic and therapeutic strategies aimed at reversing these epigenetic changes and restoring normal sperm production [33]. Overall, the interplay of genetic factors, including Y chromosome microdeletions, key gene polymorphisms, and epigenetic modifications, plays a crucial role in the pathogenesis of azoospermia. Understanding these genetic underpinnings is essential for developing effective diagnostic and therapeutic strategies for men suffering from this severe form of infertility.

## 3. The Role and Diagnostic Value of Small RNAs in Azoospermia

### 3.1. Regulatory Mechanisms and Expression Changes in miRNAs

MicroRNAs (miRNAs) are small, non-coding RNA molecules that play critical roles in the regulation of gene expression, particularly in the context of spermatogenesis and male infertility. In NOA, specific miRNAs such as miR-31-5p, miR-10a-5p, and miR-146a-5p [34] have been identified as key players in the pathological processes underlying this condition. Total RNA or small extracellular vesicles are isolated from clinical samples such as seminal plasma or testicular tissue; small RNA libraries are constructed; and deep sequencing is performed. Subsequent bioinformatic analysis involves quality control and trimming of adapters from raw sequencing reads, followed by alignment to the reference genome. The aligned reads are then annotated against reference databases, such as miRBase, to identify known miRNAs and their isoforms, and their expression levels are quantified. Finally, statistical comparisons between different patient groups are conducted using tools including DESeq2 and edgeR to identify miRNAs with significant expression changes [35]. For instance, miR-31-5p has been shown to have differential expression between azoospermic patients of different origins, suggesting its relevance in the molecular characterization of NOA [35]. Additionally, miR-30a-5p has been linked to the regulation of KDM3A, a gene implicated in male infertility, with its overexpression correlating with NOA [36]. However, it is critical to appraise the evidence supporting these candidates. For instance, while miR-31-5p has been reported to show differential expression between azoospermic patients of OA and secretory (SA) origins [35], the accurate quantification of its specific isoforms (isomiRs) is technically challenging. As Ferre et al. (2023) demonstrated, poly(A) RT-qPCR methods exhibit significant cross-reactivity between closely related miR-31-5p isomiRs, which can compromise the reliability of validation efforts and complicates the interpretation of its precise role [35]. This highlights a key methodological limitation in translating small RNA-seq discoveries into robust, quantifiable assays.

Furthermore, many miRNA biomarkers, including those frequently cited, are supported by evidence from studies with notable limitations. For example, miR-34c-5p and miR-122 have been implicated in spermatogenic processes and proposed as diagnostic biomarkers [37]. However, the pivotal study by Khadhim et al. (2023) supporting this claim was a single-center, case–control investigation with a cohort of 50 patients and 50 controls [37]. While identifying statistically significant differences, the exploratory nature and limited sample size of such studies preclude definitive conclusions about their clinical utility. The findings require validation in larger, independent, and multi-center prospective cohorts to assess generalizability and true diagnostic performance.

The emerging clinical significance of miRNA isoforms, or isomiRs, adds another layer of complexity. Dedicated bioinformatics tools (e.g., sRNAbench, SPORTS1.1) enable a more comprehensive characterization of the isomiR repertoire from sequencing data [35]. Nevertheless, as discussed above, their subsequent experimental validation remains fraught with technical challenges due to assay cross-reactivity [35]. Therefore, while isomiRs may exhibit disease-specific patterns and hold promise for more precise biomarkers, their current analysis is largely confined to the discovery phase, with significant hurdles remaining in accurate quantification and standardization. In conclusion, while miRNAs and their isoforms represent a critical and promising area of research in NOA, the current evidence for most specific candidates remains preliminary. The pathway from initial small RNA-seq discovery to validated clinical biomarker is often hindered by studies with small sample sizes, single-center designs, methodological variability in detection/validation, and a pervasive lack of independent external validation. Future research must prioritize overcoming these limitations to translate the undoubted potential of miRNAs into reliable clinical tools.

### 3.2. tsRNA and Its Potential as a Novel Biomarker

Transfer RNA-derived small RNAs (tsRNAs) encapsulated within seminal plasma extracellular vesicles have emerged as a novel class of non-invasive biomarkers for azoospermia, with particular focus on differentiating its subtypes and predicting surgical outcomes [38,39]. A pivotal study by Han et al. (2022) exemplifies this potential [40]. The researchers identified two specific tsRNAs, tRF-Val-AAC-010 and tRF-Pro-AGG-003, which demonstrated high diagnostic accuracy in distinguishing NOA from OA, with areas under the curve (AUC) of 0.96 for both [40]. More notably, tRF-Val-AAC-010 showed promise in predicting successful sperm retrieval via microdissection testicular sperm extraction (mTESE) in NOA patients, achieving an AUC of 0.89 [40]. Bioinformatic analyses further suggested these tsRNAs are involved in spermatogenesis-related pathways, linking their expression to testicular function [40].

However, the study was conducted at a single center (Xuzhou Central Hospital), and the cohort sizes were relatively small: the diagnostic analysis for distinguishing NOA from OA was based on 35 patients (20 NOA, 15 OA), and the predictive model for mTESE outcome was built on 41 NOA patients (18 Sp+, 23 Sp−) [40]. While the reported AUC values are excellent, such performance in limited, single-center cohorts is prone to overfitting and may not generalize to broader, more heterogeneous populations. The authors themselves acknowledged this key limitation, stating that their findings “should be validated in large samples from different countries or regions before clinical application” [40].

In conclusion, while tsRNAs like tRF-Val-AAC-010 represent a highly promising exploratory discovery, the current evidence supporting them remains preliminary. Their path to becoming a reliable clinical tool is contingent upon rigorous validation in large-scale, prospective, and multi-center cohorts. Future studies must also address the standardization of tsRNA isolation and quantification protocols across different laboratories to ensure reproducibility.

### 3.3. Expression and Diagnostic Limitations of piRNA

Piwi-interacting RNAs (piRNAs) are a class of small non-coding RNAs (21–35 nt) that are essential for transposon silencing and gene regulation during spermatogenesis [41,42]. Their biogenesis and function depend on PIWI-family proteins [43]. A seminal mechanistic study in mice demonstrated that a specific arginine-glycine (RG) motif in the N-terminus of the MIWI protein is indispensable for its interaction with the auxiliary factor TDRKH, efficient localization to the intermitochondrial cement, and subsequent production of pachytene piRNAs. Mutation of this RG motif led to reduced piRNA levels, chromatoid body malformation, spermatogenic arrest, and male sterility—interestingly, independent of LINE1 transposon dysregulation [44]. This work provides a robust genetic model elucidating a precise molecular mechanism by which piRNA pathway dysfunction can cause NOA.

However, a critical translational gap exists between such definitive mechanistic findings in animal models and the development of piRNAs as reliable clinical biomarkers for human azoospermia. The potential of piRNAs as diagnostic tools has been explored in human studies, with some reporting differential expression in the testicular tissue or seminal plasma of NOA patients [45]. Nevertheless, the current clinical evidence supporting specific piRNA biomarkers remains largely exploratory and faces significant limitations. The supporting studies are typically characterized by small sample sizes and single-center, case–control designs, which severely limit their statistical power and generalizability. Furthermore, there is a pervasive lack of independent external validation of these candidate piRNAs in larger, multi-center prospective cohorts. In conclusion, while the PIWI/piRNA pathway is unequivocally critical for spermatogenesis, as firmly established in model organisms [44], its direct application for the clinical diagnosis of azoospermia is not yet realized. Current human studies highlight a promising but preliminary avenue for research.

## 4. The Application of Proteomics in the Diagnosis of Azoospermia

### 4.1. Differential Expression Analysis of Seminal Plasma Proteins

The differential expression analysis of seminal plasma proteins has emerged as a pivotal area of research in understanding azoospermia, particularly in distinguishing between OA and NOA. Specific proteins such as TEX101, ECM1, and SPAG1 have been identified as potential biomarkers for this differentiation. TEX101, a testis-expressed protein, has shown significant promise in distinguishing men with abnormal semen parameters from those with normal semen parameters, with a reported sensitivity and specificity of 100% at a specific cut-off value [46]. However, while the study was well-designed and had an adequate sample size (90 cases and 90 controls), it was conducted at a single center in Southern India, which may limit the generalizability of the findings to other ethnic populations. The achievement of 100% sensitivity and specificity, though statistically significant in this cohort, is uncommon in biomarker research. This may be influenced by the relatively homogeneous study groups and the case–control design itself, which tends to overestimate diagnostic accuracy compared to real-world, prospective cohorts. Therefore, these findings require independent validation in larger, multi-center, and more diverse populations, including men with various etiologies of azoospermia, to confirm universal clinical utility and establish standardized cut-off values. Similarly, ECM1 and TEX101 have been proposed as a promising protein pair for the differential diagnosis of OA and NOA. A study reported that a seminal plasma ECM1 concentration below 2.3 μg/mL could indicate OA with 100% sensitivity and 73% specificity, and the subsequent measurement of TEX101 in ECM1-low samples improved the sensitivity for identifying NOA to 81% [47,48]. This exemplifies the potential of combined biomarker strategies. However, the clinical translation of these compelling findings faces significant hurdles. The foundational proteomic studies that identified and validated these candidates, while methodologically robust, are characterized by retrospective, single-center designs and relatively limited sample sizes [48]. Consequently, the generalizability of the reported performance metrics to broader, more diverse populations remains unconfirmed due to a lack of large-scale, multi-center external validation. The high enzymatic activity and inherent inter-individual variability of seminal plasma composition further complicate the establishment of universal, reliable diagnostic thresholds [48]. Sperm-associated antigen 1 (SPAG1) is another testis-specific protein implicated in sperm function. While its association with fertility is mechanistically plausible, the current evidence supporting its use as a standalone clinical biomarker is less developed compared to the ECM1/TEX101 combination and remains largely exploratory.

In addition to these specific markers, seminal plasma proteins such as HSPA2 [49] and LDHC have been investigated as correlates of spermatogenic status. Quantitative proteomic analysis has revealed significantly lower abundances of both HSPA2 and LDHC in the seminal plasma of men with NOA compared to fertile controls, suggesting their potential utility as non-invasive indicators of testicular function [6]. However, the study by Fietz et al. (2024), which identified HSPA2 and LDHC as among the most significantly downregulated proteins in Sertoli cell-only (SCO) phenotype patients, was a prospective but single-center investigation with a relatively limited total cohort [6]. While the study employed rigorous mass spectrometry and Western blot validation, its conclusions are derived from this constrained, single-institution sample set. Similarly, the correlation of low LDHC levels with unsuccessful sperm retrieval, while mechanistically plausible, was demonstrated within this same limited cohort. Consequently, the reported fold-changes and associations, though compelling, lack validation in independent, multi-center, and larger populations, which is a critical step in assessing generalizability and establishing reliable clinical cut-off values. Furthermore, the analytical and pre-analytical variability inherent in proteomic studies of seminal plasma poses a challenge for the standardization and reproducibility of assays measuring HSPA2, LDHC, or similar protein biomarkers [6]. In summary, proteins like HSPA2 and LDHC represent promising exploratory biomarkers that provide valuable insights into the pathophysiology of spermatogenic failure. Nevertheless, the current evidence is primarily derived from discovery-phase and initial validation studies characterized by small sample sizes and single-center designs.

### 4.2. Proteomic Technology Platforms and Their Advantages

Liquid chromatography-tandem mass spectrometry (LC-MS/MS) has emerged as a cornerstone technique in proteomics, particularly for the identification and quantification of proteins in complex biological samples [50]. This technology is particularly advantageous in the study of azoospermia, where it facilitates the analysis of testicular tissues and seminal plasma to uncover the molecular underpinnings of male infertility [6,51]. LC-MS/MS allows for the simultaneous identification of thousands of proteins, providing a comprehensive overview of the proteomic landscape. Recent studies have demonstrated its efficacy in distinguishing between OA and NOA by identifying specific protein signatures associated with each condition. For instance, a comparative proteomic analysis of formalin-fixed, paraffin-embedded (FFPE) testicular tissues identified 61 proteins with the potential to quantitatively discriminate between OA and NOA subtypes, such as SCO and hypospermatogenesis (Hyp) [51]. This study, which integrated proteomic data with transcriptomic datasets, represents a significant methodological advance in understanding the tissue-level pathophysiology of spermatogenic failure. However, the study was a single-center investigation with a discovery cohort of 27 patients and a validation cohort of 49 patients [51]. While the application of ion mobility-enhanced MS improved resolution, the generalizability of the identified protein signatures is constrained by the limited and single-center cohort. Furthermore, a key translational gap lies in determining whether these tissue-derived protein candidates are secreted and can be reliably detected in accessible biofluids like seminal plasma, a prerequisite for non-invasive diagnostics. Therefore, these findings, while mechanistically informative, remain in the discovery phase and necessitate validation in larger, multi-center studies and subsequent assay development for clinical samples.

Similarly, the strategy of using multiplexed biomarker panels is promising for improving diagnostic accuracy. Studies have evaluated combinations of testis-enriched proteins in seminal plasma. For example, HSPA2 and LDHC were reported as correlates of spermatogenic status [49], while PGK2 and ACR were proposed as predictors for successful sperm retrieval via micro-TESE, with reported high sensitivity and specificity [52]. Nevertheless, the study proposing PGK2 and ACR was a single-center, case–control study with a total of 48 NOA patients [52]. The authors explicitly noted the need for validation in larger sample sizes. The reported high diagnostic performance, while compelling, was derived from this relatively small, single-institution cohort and lacks independent external validation. The establishment of reliable clinical cut-off values requires confirmation in broader, more heterogeneous populations. In summary, while proteomic strategies have uncovered numerous candidate proteins and the logic for multi-parameter models is sound, the current evidence for specific biomarker panels is predominantly derived from exploratory, single-center studies with limited sample sizes.

### 4.3. The Value of Protein Biomarkers in Predicting Surgical Sperm Retrieval Success Rates

The identification of protein biomarkers in seminal plasma has emerged as a promising avenue for enhancing the predictive accuracy of surgical sperm retrieval success, particularly in patients with NOA. Research indicates that there are significant differences in the expression of seminal plasma proteins between patients diagnosed with SCO syndrome and those with mixed testicular atrophy (MTA) [6]. In a study comparing the proteomes of seminal plasma from fertile men and azoospermic patients, it was found that 42 proteins were significantly down-regulated in the seminal plasma of SCO patients compared to healthy controls, while only one protein showed a similar reduction in MTA patients [6]. This stark contrast suggests that the protein expression profiles in seminal plasma can serve as critical indicators of spermatogenic function, thereby aiding in the differentiation between SCO and MTA. Specifically, proteins such as HSPA2 and LDHC have been proposed as potential biomarkers for predicting the presence of sperm in the testes, which is crucial for determining the likelihood of successful sperm retrieval [6]. The ability to non-invasively assess these proteins levels could significantly reduce the number of unnecessary surgical interventions for patients who are unlikely to benefit from sperm retrieval.

Moreover, optimizing non-invasive diagnostic strategies through the integration of clinical indicators and proteomic data can enhance the management of azoospermia. Traditional diagnostic methods often fall short in accurately predicting which patients will benefit from surgical sperm retrieval, leading to a considerable burden of unnecessary procedures. By combining seminal plasma protein biomarkers with clinical assessments, healthcare providers can develop a more nuanced understanding of each patient’s condition, thereby improving decision-making processes [53]. For instance, the incorporation of proteomic profiles into routine evaluations could facilitate a more personalized approach, allowing clinicians to tailor recommendations based on the likelihood of successful sperm retrieval. This approach not only has the potential to optimize surgical outcomes but also to minimize the emotional and financial toll on patients undergoing treatment for infertility.

Furthermore, the evolving landscape of multi-omics methodologies presents an exciting opportunity to refine the predictive capabilities of non-invasive biomarkers. By leveraging genomic, transcriptomic, and metabolomic data alongside proteomic profiles, researchers can gain a comprehensive view of the biological underpinnings of azoospermia [54]. This holistic perspective may illuminate the complex interplay of genetic and environmental factors that contribute to male infertility, ultimately leading to the identification of robust biomarkers that can be reliably used in clinical practice. As artificial intelligence (AI) technologies advance, they may also play a pivotal role in integrating these diverse data streams, enhancing the predictive power of biomarkers and facilitating more informed clinical decision-making [54]. In conclusion, the exploration of protein biomarkers in seminal plasma represents a significant advancement in the field of male infertility, particularly in the context of predicting surgical sperm retrieval success.

## 5. The Relationship Between Non-Coding RNA (lncRNA) and Azoospermia

### 5.1. lncRNA IGSF11-AS1 and BVES-AS Expression Abnormalities

Long non-coding RNAs (lncRNAs) have emerged as potential regulators and biomarkers in male infertility. A 2024 case–control study by Jebur et al. investigated two testis-enriched lncRNAs, IGSF11-AS1 and BVES-AS, in the context of azoospermia [7]. The study reported that both lncRNAs were significantly downregulated in testicular tissues from 76 azoospermia patients compared to 36 fertile controls. Notably, IGSF11-AS1 demonstrated a moderate diagnostic potential with an area under the curve (AUC) of 0.84 for distinguishing patients from controls, while BVES-AS showed lower discriminative ability (AUC = 0.68) [7]. Furthermore, the expression level of IGSF11-AS1 was found to correlate with serum hormone profiles, showing a positive association with testosterone and negative associations with follicle-stimulating hormone (FSH) and luteinizing hormone (LH) [7].

However, it was a single-center, case–control investigation with a modest total sample size of 112 individuals. The cohort was drawn from a specific geographic region (Tabriz, Iran), which may limit the generalizability of the findings to other ethnic populations. Most importantly, the reported diagnostic performance and hormonal correlations lack validation in independent, external cohorts. The AUC of 0.84 for IGSF11-AS1, while suggestive of diagnostic utility, is derived from this initial, constrained dataset and requires confirmation in larger, multi-center studies. The functional mechanism by which IGSF11-AS1 influences hormone levels or spermatogenesis remains largely unexplored in this work.

In conclusion, lncRNAs such as IGSF11-AS1 represent intriguing exploratory biomarkers that link transcriptional changes with clinical phenotypes in azoospermia. The study by Jebur et al. provides a foundation for this line of inquiry [7]. Nevertheless, the current evidence is preliminary. The prognostic value of IGSF11-AS1 for predicting sperm retrieval outcomes or its utility as a non-invasive marker in seminal plasma requires rigorous investigation. For BVES-AS, the evidence for a diagnostic role is even weaker based on the available data.

### 5.2. lncRNA and miRNA Interactions and Regulatory Networks

The interplay between lncRNAs and miRNAs is increasingly recognized as a critical component of gene regulation, particularly in the context of spermatogenesis and male fertility. One notable example is the interaction between NEAT1 and miR-34a, where NEAT1 has been shown to modulate the expression of miR-34a, thereby influencing downstream target genes involved in spermatogenesis. NEAT1 acts as a competing endogenous RNA (ceRNA), sequestering miR-34a and preventing it from binding to its target mRNAs. This regulatory mechanism is particularly relevant in NOA, where dysregulation of such lncRNA-miRNA interactions may contribute to impaired spermatogenesis. Studies have demonstrated that alterations in the expression levels of NEAT1 can lead to significant changes in the levels of miR-34a, which in turn affects the expression of genes crucial for sperm development and function [55]. Furthermore, computational approaches have been developed to predict lncRNA-miRNA interactions, enhancing our understanding of these regulatory networks. For instance, the SPCMLMI method, based on structural perturbation, has shown promise in accurately predicting lncRNA-miRNA interactions, thus providing a valuable tool for elucidating the complex regulatory landscapes governing spermatogenesis [56].

In addition to NEAT1 and miR-34a, various other lncRNAs have been implicated in the regulation of spermatogenesis through their interactions with miRNAs. The molecular mechanisms underlying these interactions often involve ceRNA networks, where lncRNAs compete with mRNAs for miRNA binding. This competition can lead to the upregulation of target mRNAs when lncRNAs are present in higher concentrations, effectively modulating gene expression profiles during spermatogenesis. For example, lncRNAs such as Gm2044 have been identified as crucial regulators in spermatogenesis, acting as sponges for specific miRNAs and thereby enhancing the expression of target genes involved in germ cell development. The identification of these regulatory networks is facilitated by advanced [57] bioinformatics tools that analyze expression profiles and predict interactions, highlighting the intricate relationships between lncRNAs, miRNAs, and mRNAs in the context of male fertility.

Moreover, mechanistic studies in cellular models have elucidated specific lncRNA-miRNA interaction networks relevant to spermatogenic failure. A pivotal study demonstrated that the long non-coding RNA CASC7 is downregulated in the spermatogonia of OA patients compared to those with NOA and functions as a molecular decoy for miRNA-122-5p [58]. This competition regulates the availability of miRNA-122-5p, which itself targets the E3 ubiquitin ligase CBL. The miRNA-122-5p/CBL axis was shown to stimulate proliferation and inhibit apoptosis in a human spermatogonial stem cell (SSC) line, providing a plausible mechanism linking this lncRNA-miRNA pair to the maintenance of the spermatogenic niche [58]. However, the research focuses on testicular tissue, which is obtained invasively via biopsy. The expression and diagnostic utility of CASC7 or the CASC7/miRNA-122-5p axis in accessible biofluids like seminal plasma—a prerequisite for a non-invasive clinical test—remain completely unexplored. In conclusion, interactions such as that between lncRNA CASC7 and miRNA-122-5p exemplify the complex regulatory networks governing spermatogenesis and provide high-value exploratory insights into pathophysiology. Nevertheless, they currently represent mechanistic discoveries rather than validated clinical biomarkers.

## 6. Correlation of Gene Polymorphisms and Expression Levels with Azoospermia

### 6.1. Polymorphism Analysis of SYCP3 and TNP2 Genes

The association between genetic variations in the *SYCP3* gene and azoospermia has garnered significant attention, underscoring the genetic complexity of male infertility. Azoospermia, characterized by the complete absence of sperm in the ejaculate, affects approximately 10% of infertile men. Recent studies have identified a notable correlation, reporting that the homozygous TT genotype of a specific *SYCP3* polymorphism (T657C) is associated with an increased risk of azoospermia, suggesting a potential role in disrupted spermatogenesis [30]. The *SYCP3* gene encodes a core component of the synaptonemal complex, and its functional integrity is crucial for meiosis; thus, polymorphisms that compromise its function could directly impair sperm production.

However, the key supporting evidence comes from a single-center case–control study conducted in Tabriz, Iran, with a calculated sample size of 100 cases and 100 controls [30]. While methodologically sound in design, the generalizability of its findings to other ethnic populations may be limited. Furthermore, the use of “normospermic” men rather than confirmed fertile individuals as controls may introduce selection bias. The reported odds ratio (OR) for the TT genotype was 2.3 (95% CI: 1.0–5.3), indicating a risk factor of moderate effect size rather than a deterministic cause, and highlighting the likely contribution of other genetic and environmental factors in azoospermia. Notably, the T657C polymorphism is located in an untranslated region, and the precise molecular mechanism by which it might affect SYCP3 protein function remains to be elucidated. Despite these limitations, the observed alteration in the frequency of this *SYCP3* variant in azoospermic cohorts supports its continued investigation as a potential genetic biomarker [30]. Future studies in larger, multi-ethnic, and prospectively designed cohorts are essential to validate its clinical association and to clarify its functional impact on the meiosis.

Furthermore, the expression levels of SYCP3 and other related genes, such as DDX3Y, were found to be significantly reduced in azoospermic individuals. This reduction in mRNA expression profiles in testicular tissue suggests a direct link between genetic polymorphisms and the biological mechanisms underlying sperm production. The study highlighted that lower expression levels of these genes correlated with a higher probability of sperm retrieval in azoospermic patients, reinforcing the importance of SYCP3 as a candidate gene in infertility diagnostics [30]. This finding aligns with the notion that genetic factors, particularly those affecting meiosis and sperm development, are critical in understanding male infertility.

In addition to SYCP3, the TNP2 gene has also been implicated in male infertility, although studies have shown mixed results regarding its polymorphisms. In a population-based case–control study, the TNP2 rs199536093 GG genotype was significantly associated with idiopathic azoospermia, indicating that variations in this gene could contribute to the etiology of male infertility [59]. The findings suggest that while TNP2 polymorphisms may not universally affect all cases of male infertility, they can play a significant role in specific populations, such as the Iranian cohort studied. The association of TNP2 with male infertility underscores the need for further research to explore the implications of genetic diversity in different ethnic groups and its impact on fertility outcomes. Overall, the analysis of SYCP3 and TNP2 gene polymorphisms provides valuable insights into the genetic factors contributing to azoospermia.

### 6.2. Key Gene Expression and Its Relationship with Pathological Types

For instance, analysis of testicular biopsy tissues revealed that DDX3Y expression was significantly lower in patients with Sertoli cell-only syndrome (SCOS) than in those with hypospermatogenesis, suggesting an inverse correlation between its expression level and the severity of spermatogenic impairment [60]. Furthermore, downregulation of DDX3Y has been associated with lower sperm retrieval rates during microdissection testicular sperm extraction (mTESE), implying its potential as a molecular predictor for retrieval outcomes [29].

However, this study enrolled 12 normospermic controls and 68 NOA patients (including 40 with SCOS and 28 with maturation arrest), a relatively limited sample size from a single population source, which restricts the generalizability of the conclusions to other ethnicities and populations. Secondly, the analysis of DDX3Y expression relied entirely on testicular biopsy tissue, a sample obtained through an invasive procedure. Whether these findings can be extrapolated to non-invasive samples such as seminal plasma or serum remains unknown, which represents a major obstacle to its translation into routine clinical testing. Furthermore, although DDX3Y expression correlates with clinical phenotypes, its efficacy as an independent predictor has not been rigorously validated in prospective, large-scale cohorts, nor has it been compared with established clinical parameters such as FSH, inhibin B, or testicular volume. Therefore, although DDX3Y is an important molecule for understanding the pathological mechanisms of NOA and shows promise as a tissue-specific biomarker, the current evidence remains in the exploratory and mechanistic validation stage.

## 7. The Role of Immune Cell Infiltration and Autophagy, Ferroptosis-Related Genes in NOA

### 7.1. Expression Changes in Autophagy-Related Genes

Autophagy plays a critical role in spermatogenesis, and its dysfunction can significantly contribute to the pathophysiology of NOA. The expression of autophagy-related genes (ARGs) such as ATG3 and HSPA5 has been shown to be altered in patients with NOA, indicating a potential link between autophagy dysregulation and male infertility. For instance, a study identified 46 differentially expressed ARGs between azoospermic and control groups, suggesting that these genes are involved in crucial autophagy-associated functions and pathways essential for normal spermatogenesis [61]. Dysregulated autophagy can lead to impaired sperm cell survival and differentiation, as evidenced by the decreased expression of key autophagy markers like LC3B and Beclin-1 in azoospermic patients compared to fertile controls [61]. Furthermore, alterations in the expression of specific ARGs have been implicated in NOA, though the evidence varies in strength and clinical relevance. In patient samples, the expression of ATG4D was found to be significantly decreased in NOA and correlated with increased apoptosis in spermatogenic cells, suggesting a potential disease association [62]. However, this clinical correlation is derived from a study with a relatively limited cohort [62]. Mechanistic insights primarily come from animal models. For instance, conditional knockout (cKO) studies in mice have unequivocally demonstrated that germ cell-specific deletion of Hspa5 leads to a complete failure of spermatogenesis, positioning it as a non-redundant, cell-autonomous regulator essential for spermatogonial differentiation and meiotic initiation [63]. While this provides a robust functional validation in mice, it underscores a significant species gap; the precise role and necessity of HSPA5 in human spermatogenesis remain to be directly confirmed. At the cellular level, a competing ceRNA network involving the downregulation of lncRNA LINC01527, consequent upregulation of miR-766-3p, and suppression of its target ATG7 has been proposed as a mechanism contributing to autophagy dysregulation in a human cell model of NOA [64]. This represents a plausible in vitro mechanism but requires validation in primary human testicular tissues. The therapeutic potential of modulating autophagy is supported by preclinical studies. The antioxidant edaravone was shown to ameliorate spermatogenic defects in a mouse model of NOA, partly by restoring autophagic flux and reducing oxidative stress [65]. Nevertheless, this pharmacological effect, while promising, is confined to an animal model and its efficacy and safety in humans are unknown.

Recent bioinformatics analyses have attempted to consolidate these findings by screening transcriptomic datasets to nominate specific ARGs as diagnostic biomarkers for NOA. One such study identified a panel of four genes—ATP6V1E2, UBQLN2, FYCO1, and ITPR1—with high diagnostic AUC values in silico [66]. Critically, however, the authors of that study explicitly acknowledged its limitations, including the retrospective nature of the public data, the need for validation in larger cohorts, and, most importantly, the fact that the diagnostic performance and biological relevance of these computationally prioritized genes lack experimental confirmation in independent clinical samples [66].

In conclusion, while dysregulation of ARGs like ATG4D, HSPA5, and ATG7 is mechanistically linked to spermatogenic failure in models and shows correlative changes in patient tissues, the current evidence is stratified. Robust functional data exist primarily in mice, while human clinical evidence is often correlative and from studies with limited sample sizes. Computational biomarker candidates, though intriguing, are preliminary. Therefore, targeting autophagy represents a compelling exploratory avenue for understanding NOA pathophysiology. However, its translation into validated clinical diagnostics or therapies is not imminent and is contingent upon large-scale prospective validation of candidate ARGs in human cohorts and functional studies in relevant human cellular systems to bridge the gap between animal models and human disease.

### 7.2. Ferroptosis-Related Genes and Testicular Cell Function

Ferroptosis, a form of regulated cell death characterized by iron-dependent lipid peroxidation, has emerged as a significant player in the pathophysiology of NOA. The involvement of ferroptosis-related genes such as GPX4 and HMOX1 in testicular cell function highlights the complex interplay between iron metabolism and spermatogenesis. GPX4, a critical antioxidant enzyme, protects cells from lipid peroxidation, and its downregulation has been associated with increased susceptibility to ferroptosis in various cell types, including Leydig cells. Studies have shown that exposure to environmental toxins such as bisphenol A (BPA) can induce ferroptosis in testicular cells, leading to impaired spermatogenesis and reduced fertility [67]. The mechanism involves the disruption of iron homeostasis and the accumulation of reactive oxygen species (ROS), which overwhelm the cell’s antioxidant defenses. For instance, BPA exposure not only triggers apoptosis but also promotes ferroptosis by altering lipid metabolism, which is crucial for maintaining cellular integrity during spermatogenesis [68]. Furthermore, the expression of ferroptosis-related genes is significantly altered in response to oxidative stress, indicating that the ferroptosis pathway is intricately linked to testicular cell function and fertility outcomes.

The interaction between immune cell infiltration and the mechanisms of cell death, including ferroptosis, further complicates the landscape of NOA. Inflammatory responses within the testis can exacerbate oxidative stress, leading to increased ferroptosis in testicular cells. For example, the infiltration of immune cells can release pro-inflammatory cytokines that not only induce oxidative stress but also alter the expression of ferroptosis-related genes, creating a feedback loop that exacerbates testicular damage [69]. This interplay suggests that the immune microenvironment plays a crucial role in modulating ferroptosis and, consequently, spermatogenesis. Moreover, the relationship between iron metabolism and immune cell function is pivotal, as iron is essential for both spermatogenesis and the activation of immune cells. Dysregulation of iron homeostasis can lead to excessive lipid peroxidation and ferroptosis, resulting in impaired testicular function and fertility. Overall, the evidence points to a critical need for further research into the molecular mechanisms governing ferroptosis in testicular cells, as well as the potential for therapeutic interventions that could restore normal spermatogenesis and improve fertility in men suffering from NOA.

## 8. Progress in the Application of Imaging Techniques in the Diagnosis of Azoospermia

### 8.1. Ultrasound and Magnetic Resonance Imaging Techniques

The application of advanced imaging techniques, such as diffusion tensor imaging (DTI) and fiber tractography (FT), has shown promising potential in characterizing microstructural alterations in azoospermia. However, while these imaging modalities offer valuable information for etiological differentiation, their utility in reliably predicting sperm retrieval outcomes prior to microdissection testicular sperm extraction (mTESE) remains limited. This limitation is underscored by a recent prospective study employing epididymal DTI/FT [16]. Although the study reported significant microstructural changes in the epididymides of men with NOA compared to controls, its DTI parameters were unable to differentiate between histologic phenotypes of NOA or to predict sperm retrieval success (*p* > 0.05). It is crucial to critically appraise this evidence: the study was conducted at a single center with a relatively small sample size (22 NOA patients and 15 controls), and the authors explicitly acknowledged the need for validation in larger cohorts. Therefore, the current negative predictive finding, while informative, stems from a preliminary investigation. The inability to predict outcomes may be attributed to the complex, multifactorial nature of sperm retrieval success, which may not be fully captured by a single imaging biomarker or a study of this scale. Consequently, while DTI/FT provides novel pathophysiological insights, its role as a standalone, reliable preoperative predictor for mTESE is not yet established and requires confirmation through larger, multi-center studies [16].

In addition to DTI and fiber tractography, the correlation between imaging parameters and the histological types of NOA has been investigated. A study analyzing the relationship between MRI parameters and testicular histology found significant differences in ADC values among various histological phenotypes of NOA, including Sertoli cell-only syndrome and maturation arrest [70]. These findings suggest that MRI could potentially aid in the classification of NOA subtypes, which is essential for tailoring treatment strategies. Moreover, the ability to correlate imaging findings with histological outcomes could improve the prognostic capabilities of imaging techniques, allowing for more informed decision-making regarding surgical interventions such as microdissection testicular sperm extraction (mTESE). Nonetheless, the current limitations of imaging techniques in predicting sperm retrieval success necessitate ongoing research to explore their full diagnostic potential.

The integration of imaging techniques with traditional diagnostic methods has the potential to enhance the overall evaluation of azoospermia. For instance, combining transrectal ultrasound with MRI has been shown to improve the detection of obstructive causes of azoospermia, such as ejaculatory duct obstruction [71]. This multimodal approach not only facilitates a comprehensive assessment of the male reproductive tract but also aids in differentiating between obstructive and non-obstructive causes of azoospermia. The ability to accurately diagnose the underlying etiology is crucial for determining appropriate treatment options and improving fertility outcomes. Furthermore, the application of high-frequency ultrasound has been explored for assessing seminiferous tubules, providing additional insights into testicular pathology in azoospermic patients [72].

### 8.2. Non-Invasive Advantages and Limitations of Imaging Techniques

The current clinical application of imaging techniques in the diagnosis of azoospermia highlights their non-invasive advantages, primarily through modalities such as MRI and ultrasound. Scrotal MRI, particularly using diffusion tensor imaging (DTI) and fiber tractography, has emerged as a promising tool in evaluating NOA. Studies have demonstrated that DTI can reveal lower fractional anisotropy in men with NOA compared to normal individuals, indicating potential abnormalities in the epididymis that correlate with infertility [16]. Furthermore, MRI parameters such as the apparent diffusion coefficient (ADC) and magnetization transfer ratio have been shown to provide useful insights into spermatogenesis status and can help differentiate between various etiologies of azoospermia [70]. The non-invasive nature of these imaging techniques allows for comprehensive assessments without the need for surgical interventions, thus reducing patient discomfort and risk of complications. However, despite these advantages, limitations persist. For instance, while MRI can provide valuable information regarding testicular histology and sperm retrieval outcomes, it is not always predictive of sperm presence before procedures like mTESE [17]. Additionally, the high cost and limited availability of advanced imaging technologies can restrict their widespread use in clinical practice.

Looking towards the future, the development trends in imaging technology for azoospermia diagnosis are likely to focus on enhancing resolution and predictive capabilities. Emerging techniques such as high-frequency ultrasound (HFUS) and advanced machine learning algorithms for image analysis show promise in improving diagnostic accuracy [72,73]. HFUS has demonstrated potential in assessing seminiferous tubule structure and correlating findings with histopathological results, which could further refine the diagnostic process for azoospermia [1]. Moreover, the integration of artificial intelligence in imaging analysis could facilitate real-time assessments, allowing for quicker and more accurate diagnoses. For instance, deep learning models have been developed to predict histological outcomes based on ultrasound images, potentially reducing the need for invasive biopsies [73].

## 9. Application of Machine Learning and Multi-Factor Models in Azoospermia Diagnosis

### 9.1. Machine Learning Algorithms for Key Predictive Factor Selection

Machine learning (ML) has been applied to integrate clinical and biomarker data to improve the prediction of NOA. A recent study developed a diagnostic nomogram by employing multiple ML algorithms on a retrospective cohort of 352 azoospermic patients [74]. The model identified FSH and semen pH as positive predictors, while inhibin B (INHB) and mean testicular volume (MTV) were negative predictors. A comparative analysis of nine ML methods found the Gradient Boosting Decision Tree algorithm to be superior, achieving an area under the curve (AUC) of 0.974, outperforming other models like Random Forest (AUC = 0.953) in this specific cohort [74]. The final nomogram, constructed with the four key features, demonstrated an exceptionally high AUC of 0.984 in the training set and 0.976 in the internal validation set, providing clinically actionable cut-off values (e.g., FSH > 7.50 IU/L, INHB > 43.45 pg/mL) [74].

However, the model was developed and validated solely on a single-center, retrospective dataset, with the internal validation set (*n* = 108) derived from the same patient pool as the training data. The authors explicitly acknowledged the lack of external validation as a key constraint [74]. The implausibly high and nearly identical AUCs between training and validation sets (ΔAUC = 0.008) raise significant concerns about overfitting, potential data leakage, or a non-independent validation split. Consequently, the reported performance metrics are likely an optimistic estimate specific to the original cohort and may not generalize to broader, more diverse populations. The study exemplifies the common challenge in medical ML where impressive internal validation is not synonymous with clinical readiness. Nevertheless, the findings remain preliminary and hypothesis-generating. The critical absence of independent external validation means the model cannot yet be considered a robust clinical tool. Future research must prioritize the development and strict validation of ML models in large, multi-center, prospective cohorts to assess their true generalizability and clinical utility before integration into diagnostic pathways.

### 9.2. Construction of Predictive Models and Clinical Applications

The development of predictive models for NOA aims to personalize patient care, primarily by estimating the likelihood of successful sperm retrieval (SRR) or subsequent clinical pregnancy. Models predicting SRR often integrate routine clinical parameters. For instance, one study used binary logistic regression on retrospective data from 200 NOA patients to identify six predictors for successful micro-TESE, including anti-Müllerian hormone, inhibin B, and clinical etiology, achieving an area under the curve (AUC) of 0.720 [75]. However, this model is constrained by its development on a relatively limited, single-center retrospective cohort (*n* = 200). The reported AUC, while reasonable, reflects moderate discriminative ability, and the model’s performance lacks confirmation in an independent external cohort, limiting assessment of its generalizability.

Beyond SRR, other models target the ultimate clinical endpoint of pregnancy. A study developed a clinical pregnancy prediction model for couples undergoing ICSI with surgically retrieved sperm [76]. Utilizing data from 453 couples, the model incorporated eight predictors, including azoospermia type, testicular volume, and key male/female hormonal profiles, achieving a high AUC of 0.891 in the development set and 0.866 in the internal validation set, with good calibration [76]. This represents a more comprehensive, couple-centric approach. Nevertheless, the authors explicitly acknowledged the model was developed and validated within a single center. They identified the need for validation in different clinical populations from other centers before the model can be widely adopted, highlighting a common and critical limitation of external validity. In conclusion, while predictive modeling holds significant promise for personalizing NOA management, the current evidence base is heterogeneous and predominantly exploratory. Existing models are almost exclusively derived from single-center, retrospective studies of varying sample sizes. A systematic comparison of these diagnostic and prognostic strategies is presented in Table 1.

**Table 1:** Comparison of Diagnostic and Prognostic Strategies for Non-Obstructive Azoospermia (NOA).

Assessment Dimension/Methodology	Primary Advantages	Limitations/Challenges
Traditional Clinical Diagnostics (Testicular biopsy [6], Hormones [77], Ultrasound [16,17])	Gold standard for histological diagnosis (biopsy). Provides direct anatomical (ultrasound) and endocrine (FSH, Inhibin B) assessment.	Invasive (biopsy), risk of complications. Poor predictors of sperm retrieval success. Hormonal levels can be non-specific.
Single Biomarker Detection [47,49]	Minimally or non-invasive (blood, semen). Lower cost compared to multi-omics. Potential for high specificity.	Lack of standardization in detection methods. Variable stability and specificity. Often insufficient to capture disease complexity alone.
Multi-omics Biomarker Integration [52,78,79]	Comprehensive, systems-level view. Improved diagnostic accuracy by combining multiple markers. Reveals pathophysiological insights.	High cost and complexity of analysis. Challenging data integration. Requires validation in large cohorts for clinical translation.
Advanced Imaging Techniques (Scrotal MRI [16], DTI [16], HFUS [80])	Completely non-invasive. Provides structural and functional information (microstructure, diffusion). Correlates with histology.	High cost and limited availability. Operator-dependent. Currently limited predictive value for sperm retrieval success.
Machine Learning/Multi-Factor Predictive Models [81]	Handles complex, multidimensional data (clinical + biomarkers + imaging). Personalized risk prediction. High predictive performance (AUC).	Requires large, high-quality datasets for training. Risk of overfitting. “Black box” nature can reduce interpretability.

## 10. The Comorbidity Mechanisms and Molecular Associations of Azoospermia with Other Diseases

### 10.1. Common Signaling Pathways and Key Genes

The interplay between common signaling pathways and key genes is crucial in understanding the pathogenesis of azoospermia, particularly the interleukin-17 (IL-17) signaling pathway. Recent studies have highlighted the role of IL-17 as a pivotal cytokine in the inflammatory response associated with male infertility. Elevated levels of IL-17 have been observed in patients with azoospermia and oligozoospermia, suggesting its potential role as a biomarker for these conditions. For instance, a study involving 93 participants demonstrated that IL-17 levels were significantly higher in azoospermic patients compared to controls, indicating a link between IL-17 and impaired spermatogenesis [82]. Furthermore, the integration of machine learning methods has identified key genes such as GLO1 and GPR135 that are differentially expressed in azoospermia cases. These genes may serve as potential biomarkers for diagnosis and therapeutic targets. GLO1, for example, is involved in the detoxification of methylglyoxal, a byproduct of glycolysis that can induce cellular stress; thus, its dysregulation could contribute to testicular dysfunction and infertility [80]. Additionally, GPR135, a G protein-coupled receptor, has been implicated in various reproductive processes, and its altered expression may reflect the underlying pathophysiology of azoospermia.

Moreover, the impact of comorbid diseases on male reproductive function cannot be overlooked. Conditions such as obesity, diabetes, and infections have been linked to alterations in the signaling pathways involved in spermatogenesis. For instance, inflammation caused by comorbidities can exacerbate the dysregulation of cytokines like IL-17, further impairing sperm production and quality. The inflammatory microenvironment may hinder the normal functioning of Sertoli cells, which are essential for nurturing developing sperm cells. This highlights the need for a comprehensive understanding of how systemic health influences reproductive outcomes in men. In summary, the common signaling pathways, particularly the IL-17 pathway, along with the identification of key genes through machine learning methodologies, provide valuable insights into the pathogenesis of azoospermia.

### 10.2. Application of Integrative Bioinformatics Analysis

Integrative bioinformatics analysis, leveraging data from diverse diseases and conditions, has emerged as a tool for generating hypotheses about the complex pathogenesis of NOA. This approach often employs weighted gene co-expression network analysis (WGCNA) and machine learning algorithms to mine publicly available transcriptomic datasets. For instance, such analyses have been used to explore potential shared mechanisms between COVID-19 and NOA, identifying overlapping pathways and hub genes [80]. More commonly, these methods are applied to NOA-specific datasets to nominate dysregulated pathways and candidate biomarkers. Studies have highlighted the significant dysregulation of genes associated with ferroptosis, a form of iron-dependent programmed cell death, in NOA, linking oxidative stress and lipid peroxidation to spermatogenic failure [77,83]. Similarly, bioinformatic screens have implicated ARGs and specific immune infiltration patterns in the testicular microenvironment of NOA patients [81]. However, many analyses, such as those identifying ferroptosis-related gene signatures from the GSE145467 dataset (*n* = 20) [77] or autophagy-related hubs from combined datasets [81], remain primarily in silico predictions. The authors of these studies explicitly acknowledge limitations including “small sample size” and the need for “experimental verifications” [77,81].

A more robust tier of evidence comes from studies that couple bioinformatic discovery with experimental validation in patient samples. The research on ferroptosis in iNOA represents this approach [83]. It began with bioinformatic screening of 63 testicular samples, identified four hub genes (GPX4, DUSP1, SLC2A8, HSD17B11), and then validated their dysregulation at the RNA and protein levels in an independent clinical cohort. Furthermore, it provided morphological and biochemical evidence for the ferroptosis phenotype in iNOA testes [83]. Nevertheless, the cohort was still a single-center collection of 28 iNOA patients and 10 controls. The authors noted the “amount of sample was low” for some assays and concluded that their findings “should be validated in a larger iNOA cohort” [83]. This highlights a pervasive issue: the lack of large-scale, multi-center, prospective external validation for virtually all computationally derived biomarkers, which prevents an assessment of their true diagnostic generalizability and clinical utility.

**Figure 2:**
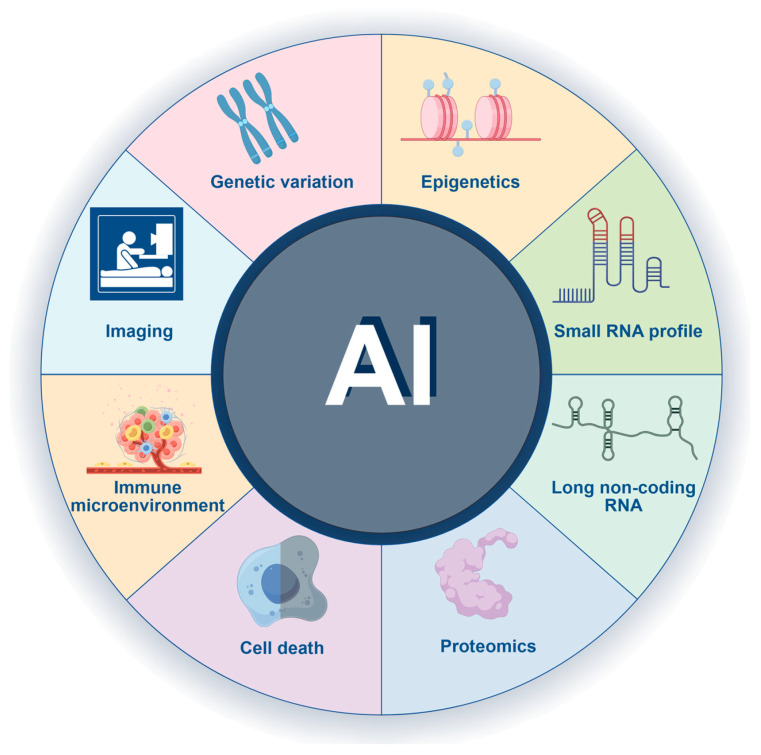
Integrated Application of Artificial Intelligence in Multi-Omics Research on Azoospermia (created with Figdraw, www.figdraw.com). This schematic diagram (circular chart) illustrates how Artificial Intelligence (AI) serves as a core analytical tool to integrate and interpret the multi-dimensional, multi-layered biological data of azoospermia, particularly Non-Obstructive Azoospermia (NOA), aiming to elucidate its complex etiology and discover novel biomarkers. The “AI” at the center represents artificial intelligence algorithms. The eight surrounding sectors represent the key data types and research directions integrated by AI in such studies, including: Genetic Variation, Epigenetics, Small RNA Profiles, Long Non-coding RNAs (lncRNAs), Proteomics, Cell Death, Immune, and Microenvironment. This figure emphasizes the paradigm shift from traditional single-biomarker analysis towards a systematic research framework utilizing AI to integrate genomic, transcriptomic, proteomic, and microenvironmental information, with the goal of achieving more precise disease subtyping, diagnosis, and the discovery of therapeutic targets.

Integrative bioinformatics has emerged as a powerful framework for deciphering the complex mechanisms of azoospermia by fusing multi-omics data, nominating numerous candidate genes and non-coding RNAs for NOA. Nonetheless, the current evidence landscape remains predominantly exploratory, characterized by studies with limited sample sizes, retrospective designs, and a heavy reliance on single-center data. A conceptual overview of this multi-omics integration strategy is depicted in Figure 2. To translate these preliminary discoveries into mechanistic understanding and clinical applications, future studies must prioritize validation in large, prospective cohorts.

## 11. Current Status and Challenges of Clinical Applications of Seminal and Serum Biomarkers

### 11.1. Advantages and Clinical Significance of Non-Invasive Biomarkers

The exploration of non-invasive biomarkers for diagnosing azoospermia has gained significant traction in recent years, primarily due to the need for less invasive diagnostic procedures that can accurately predict sperm retrieval outcomes. Among these biomarkers, the detection of seminal plasma extracellular vesicle RNA, proteins, and serum non-coding RNAs (ncRNAs) has emerged as a promising avenue. Studies have shown that specific tRNA-derived small RNAs (tsRNAs) found in seminal plasma can serve as effective biomarkers for differentiating between NOA patients with successful sperm retrieval (Sp+) and those without (Sp−) [41]. For instance, the tsRNA tRF-Val-AAC-010 demonstrated high predictive accuracy with an area under the curve (AUC) of 0.89, indicating its potential utility in clinical settings to assess the likelihood of sperm presence before surgical intervention. Furthermore, the ability to analyze proteins in seminal plasma has also shown promise, with proteins such as TEX101 and ECM1 being identified as significant biomarkers for diagnosing azoospermia. The combined detection of these proteins has been associated with improved diagnostic sensitivity and specificity, underscoring the importance of utilizing multiple biomarkers in tandem for enhanced diagnostic accuracy [48].

Moreover, integrating multiple non-invasive biomarkers holds promise for refining azoospermia diagnosis and prognostication. Research has explored the combined utility of molecular biomarkers in seminal plasma and clinical parameters analyzed by advanced computational methods. For instance, a study profiling seminal plasma microRNAs identified hsa-miR-34c-5p as significantly downregulated in patients with NOA compared to fertile controls, reporting high AUC values (0.979–0.987) for distinguishing between different histopathological subtypes [84]. Concurrently, machine learning models integrating routine clinical data—such as FSH, inhibin B, mean testicular volume, and semen pH—have demonstrated the ability to predict NOA with high reported AUCs (0.984 in training, 0.976 in validation) in a dedicated cohort [74]. However, the study on hsa-miR-34c-5p, while identifying a compelling candidate, is limited by its development and validation within a single-center cohort. The initial sequencing phase included a modest sample size (13 NOA patients, 7 controls), and the subsequent validation cohort, while larger, remains from the same institution [84].

### 11.2. Limitations of Existing Biomarkers and Future Needs

Despite the identification of numerous promising candidate biomarkers across diverse categories—as systematically summarized in Table 2—their translation into routine clinical practice is impeded by a constellation of shared and fundamental limitations. A critical synthesis of the evidence reveals that the field remains predominantly in the exploratory phase. The most pervasive issues include the reliance on studies with small sample sizes, retrospective and single-center designs, and a near-universal lack of independent, external validation in large, multi-center prospective cohorts. This severely limits the generalizability of reported performance metrics (e.g., high AUC values) and prevents an accurate assessment of their real-world diagnostic utility. Furthermore, methodological heterogeneity in sample processing, assay platforms, and data analysis compromises reproducibility and complicates the comparison of results across different laboratories. For many biomarkers, especially non-coding RNAs, the evidence is often correlative, with a significant gap between biomarker discovery and the elucidation of their precise functional mechanisms in human spermatogenic failure.

To overcome these barriers and navigate the path from discovery to application, future research must embrace a more rigorous and integrated paradigm. Priority must be given to prospective, large-scale, multi-center validation studies to confirm diagnostic accuracy and establish clinically robust cut-off values. Concurrently, efforts towards standardizing pre-analytical and analytical protocols are essential. The ultimate goal should be to move beyond single-omics approaches and leverage multi-omics integration—combining genomic, transcriptomic, proteomic, and clinical data—to capture the complex pathophysiology of azoospermia. Artificial intelligence will be crucial in analyzing these complex datasets and building clinically actionable models.

**Figure 3:**
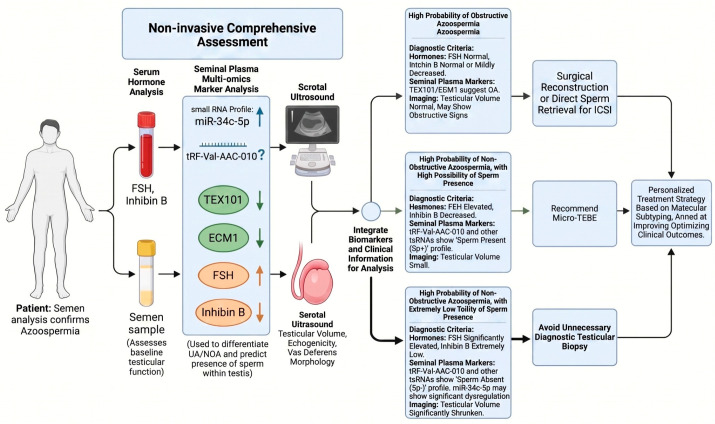
An integrated multi-omics diagnostic and clinical decision-making pathway for azoospermia (created with Figdraw, www.figdraw.com). This flowchart illustrates a proposed clinical workflow for the diagnosis and management of azoospermia by integrating traditional clinical parameters with emerging multi-omics biomarkers. The process begins with a Non-invasive Comprehensive Assessment, encompassing serum. Arrows indicate the direction of change: “up” represents upregulation/increase, “down ” represents downregulation/decrease. The question mark (?) indicates unknown or controversial findings.

This integrative vision is conceptually embodied in Figure 3, which proposes a multi-omics informed diagnostic and clinical decision-making pathway. This pathway illustrates how traditional parameters can be combined with validated molecular biomarkers from seminal plasma to stratify patients into distinct management categories: those with probable OA, NOA with a high likelihood of sperm retrieval, and NOA with a very low likelihood. The objective of such an integrated approach is to translate biomarker discoveries into clinical algorithms that personalize patient management, optimize treatment selection, and ultimately avoid unnecessary diagnostic or therapeutic procedures.

The discovery and validation of specific molecular signatures, such as distinct profiles of microRNAs (miRNAs), tRNA-derived small RNAs (tsRNAs), and proteins, are revolutionizing our approach. These biomarkers do more than just offer a non-invasive alternative to invasive surgical sperm retrieval for diagnostic purposes; they provide a window into the underlying pathological states of the testis, enabling a more precise classification of azoospermia types. Furthermore, the integration of these molecular data with advanced clinical imaging techniques creates a powerful synergistic effect, yielding a more comprehensive evaluation of the reproductive tract (Table 3).

## 12. Conclusions

Azoospermia, characterized by the complete absence of sperm in the ejaculate, represents a profound challenge in male reproductive medicine due to its multifactorial pathogenesis, which involves a complex and still not fully elucidated interplay of genetic predispositions, molecular dysfunctions, immunological factors, and cellular defects. This intricate etiology underscores the critical need for a deeper, more holistic understanding of the underlying mechanisms to pave the way for effective diagnostic and therapeutic interventions. As highlighted in this review, the field has witnessed significant progress, particularly in the move towards non-invasive diagnostic methodologies that leverage molecular biomarkers found in seminal plasma or blood. Perhaps most promising is the application of machine learning algorithms to analyze these complex, multifactorial datasets, which enhances diagnostic precision and paves the way for personalized prognostic assessments and treatment strategies. Therefore, the future of azoospermia management lies in a deeply integrated approach. Prioritizing the large-scale clinical validation and standardization of these novel biomarkers is the essential next step, which must be followed by the systematic exploration of multi-omics data integration—combining genomics, transcriptomics, proteomics, and metabolomics—to construct a holistic disease model that will ultimately unlock new therapeutic targets and significantly improve clinical outcomes for affected individuals.

In conclusion, the shift towards molecular biomarker-based diagnostic methods holds immense promise for the management of azoospermia. By reducing reliance on traditional invasive techniques, these advancements pave the way for more precise and personalized care for patients facing this challenging condition. As we continue to unravel the complexities of azoospermia through ongoing research and technological innovation, we stand on the brink of a new era in reproductive medicine, one that prioritizes patient-centered approaches and fosters the restoration of fertility in affected individuals. The collaborative efforts of researchers, clinicians, and technologists will be essential in driving this progress forward, ultimately enhancing the quality of life for countless patients struggling with azoospermia and its associated challenges.

## Figures and Tables

**Table 2:** Summary of Key Diagnostic and Prognostic Biomarkers in Azoospermia.

Biomarker Category	Specific Examples	Source Sample	Association with AZO Type/Pathology	Potential Clinical Utility/Function
Small RNAs: miRNA	miR-31-5p, miR-10a-5p, miR-146a-5p	Seminal plasma, Testis	Differential expression in NOA vs. normal; linked to pathogenesis.	Regulate spermatogenesis-related genes; potential non-invasive diagnostic markers [37].
	miR-30a-5p		Overexpression correlates with NOA.	Regulates KDM3A, a gene implicated in male infertility [38].
	miR-34c-5p, miR-122		Implicated in spermatogenic processes.	Modulate genes in spermatogenesis; part of regulatory networks [40].
Small RNAs: tsRNA	tRF-Val-AAC-010, tRF-Pro-AGG-003	Seminal plasma extracellular vesicles	Distinguish OA from NOA; predict sperm retrieval (Sp+ vs. Sp−) in NOA.	Non-invasive biomarkers for differentiating AZO types and predicting mTESE outcome [42].
Small RNAs: piRNA	Various piRNAs	Testis, Seminal fluid	Dysregulation linked to NOA; distinct profiles in infertility subtypes.	Crucial for germ cell development and transposon silencing; diagnostic potential under investigation [43,44,45].
Proteins	TEX101, ECM1, SPAG1	Seminal plasma	Differentiate OA from NOA; correlate with ART outcomes.	Non-invasive diagnostic biomarkers for AZO classification [47].
	HSPA2, LDHC	Seminal plasma	Correlate with spermatogenic status; predict sperm retrieval success in NOA.	Indicators of spermatogenic function and predictors for surgical sperm retrieval [50,51].
Long Non-coding RNA (lncRNA)	IGSF11-AS1	Serum, Tissue	Downregulated in azoospermia; correlates with testosterone and FSH levels.	Hormonal regulation; may serve as a biomarker for spermatogenic health [7].
	BVES-AS		Dysregulated in azoospermia.	Involved in cell adhesion/angiogenesis; implications in reproductive health [7].
	NEAT1		Interacts with miR-34a; dysregulated in NOA.	Acts as a ceRNA, influencing spermatogenesis-related gene expression [56].
Gene Polymorphisms and Expression	SYCP3 (C allele, TT genotype)	Blood, Testis	Associated with increased likelihood of azoospermia.	Disrupts synaptonemal complex formation; potential genetic biomarker [32].
	TNP2 (rs199536093 GG)	Blood	Associated with idiopathic azoospermia in specific populations.	Contributes to the genetic etiology of male infertility [60].
	DDX3Y expression	Testis	Reduced expression in NOA, especially in SCOS; correlates with sperm retrieval rates.	Crucial for spermatogenesis; expression level as a diagnostic and prognostic biomarker [30,61].
Imaging Indicators	DTI parameters (FA, ADC)	MRI scan	Altered epididymal microstructure in NOA; correlates with histology.	Non-invasive assessment of epididymal function and testicular histology classification [16,74].

**Table 3:** Clinical Translation Readiness of Azoospermia Biomarkers.

Specific Biomarker	Current Validation Status	Key Advantages/Findings	Key Limitations
tRF-Val-AAC-010 [41]	Exploratory, single-center validation	High AUC for distinguishing NOA from OA; potential for predicting mTESE outcome	Small sample size, single-center study, lacks independent cohort validation
miR-31-5p [35], miR-146a-5p [34]	Differential expression reported in multiple studies; partially validated in independent cohorts	Stable in seminal plasma/tests; mechanistically linked to spermatogenesis	Lack of assay standardization; no unified cut-off values
TEX101 [46,48]	Validated in an independent cohort	High sensitivity and specificity reported; defined cut-off value	Study in a single ethnic population; lack of prospective application data
SYCP3 gene polymorphism [30]	Single-center case–control study	Provides etiological insight; OR = 2.3	Moderate effect size; unknown mechanism; lacks multi-population validation
Nomogram based on FSH, INHB [74]	Internal validation performance; lacks external validation	Integrates multiple parameters; decision curve analysis shows potential clinical utility	Single-center retrospective data; potential overfitting; no external validation

## Data Availability

Data sharing is not applicable to this article as no new data were created or analyzed in this study. All information discussed is sourced from the publications cited in the reference list.
